# Integration of a Tobacco Treatment Specialist into Primary Care: Perception from Multidisciplinary Team

**DOI:** 10.1155/2022/9330393

**Published:** 2022-06-18

**Authors:** Amanda F. Meyer, Abby Cervenka, Lacey Lammers, Joseph Furst

**Affiliations:** Mayo Clinic-Southeast Primary Care, 4452 Canal Pl SE, Rochester, MN 55904, USA

## Abstract

**Background:**

Tobacco continues to be on the leading cause of avoidable death. Primary care practices are ideal locations to provide tobacco cessation visits. Tobacco treatment specialists are trained individuals with expertise in providing medication and counseling management to patients to help with tobacco cessation.

**Purpose:**

The purpose of this study was to examine the integration of a tobacco treatment specialist into the primary care setting and the perception of this role from the multidisciplinary team.

**Method:**

We conducted an electronic cross-sectional survey to evaluate awareness and perception of the integration of a tobacco treatment specialist into a primary care facility that is part of a large Midwestern tertiary healthcare center. The sample for this study included all the primary clinic staff that directly work with patients and included licensed practical nurses, registered nurses, physician assistants, certified nurse practitioners, and medical doctors.

**Results:**

55% (*n* = 22) of staff had utilized the tobacco treatment specialist with direct patient care. Reasons for using the specialist was for referral for follow-up tobacco cessation visit (54%), curbside consultations (21%), medication management (21%), and other reasons (5%). The majority of staff strongly agreed that utilizing the TTS was valuable.

**Conclusion:**

This study reinforced the positive impact a tobacco treatment specialist can have being integrated into the primary care practice from the perception of the multidisciplinary team.

## 1. Introduction

Tobacco use continues to be one of the leading causes of avoidable death as tobacco use impacts nearly every system of the body [1]. Yet, 13.7% of all adults in the United States are current cigarette smokers [2]. Primary care providers are in the prime position to provide tobacco cessation treatment to patients, but multiple barriers impact this ability such as lack of time and decreased knowledge to provide evidence-based medication and counseling. Implementing a tobacco treatment specialist into the primary care setting has the potential to provide resources to staff and conduct tobacco cessation visits in the convenience of the patient's own primary care practice.

Tobacco use causes significant health issues to those who smoke, and on average, smokers suffer with more health problems such as increased infections, periodontals or gum disease, all types of cancers, Crohn's disease, and rheumatoid arthritis. In addition, smokers on average die a decade earlier than nonsmokers [1]. If smokers are able to abstain from smoking long-term, there are significant improvements to health outcomes. On average, most smokers have tried 2-3 times to quit, and around 40.1% of smokers report attempts to quit each year. This correlates with smokers who make multiple quit attempts and think frequently about quitting smoking [3]. This desire to quit is an opportunity for providers to have discussions with patients.

Recently, the Surgeon General's report explained evidenced-based clinical interventions to help increase the number of those who successfully quit smoking. These interventions are comprehensive, and include counseling and medications. This report reinforced that we need to increase access to treatments and resources for quitting [4]. Primary care is in a unique position to provide these services because they allow a multidisciplinary approach to care with the patient. In addition, close follow-up in this setting allows increased support to prevent relapse. Guidelines recommend that every patient encounter should include obtaining a smoking status history followed by the opportunity to receive smoking cessation counseling. A study that reviewed patient perspectives on tobacco use treatment in primary care found that patients desired from their providers consistent, honest, and practical discussions and actions when engaging in smoking cessation counseling [5]. Integrating smoking cessation strategies, education, and interventions should be an important component of a primary care medical practice. Individuals who are counseled to quit smoking by their providers are 1.6 times more likely to stop smoking compared to those patients not receiving smoking cessation advice [6]. Assessing tobacco use, readiness to quit, and interventions are often not completed due to numerous barriers found in the primary care setting [7]. These barriers include but are not limited to time constraints, money issues along with treatment reimbursement, provider competence, comfort with tobacco treatment, and personal attitudes by the providers in regard to nicotine dependence [8]. Tobacco treatment specialists have the training and ability to provide these interventions in the primary care setting.

Tobacco treatment specialists (TTS) are professionals who have skills, proficiency, and training to provide effective comprehensive evidence-based interventions for tobacco dependence across a range of specialities [9]. These specialists have been provided standardized education from a rigorous training program with national certification. The unified training focuses on core competencies which include tobacco-dependence knowledge and education, counseling skills, assessment interviews, treatment planning, pharmacotherapy, relapse prevention, diverse and specific health issues, documentation and evaluations, professional resources along with development, and specific law and ethics in regard to providing tobacco-dependence treatment [9]. Since these specialists have the ability to provide highly effective specialty care in various settings, they have the potential to have a large impact in primary care.

A systematic review found that colocation of specialty care within primary care practice settings was associated with reduced appointment wait times, reduced costs, improvement in patient measurable outcomes, and improvements in quality of life [10]. Most importantly, having specialty care within the primary care practice increased patient and provider satisfaction [10].

The purpose of this study was to examine the integration of a tobacco treatment specialist into the primary care setting and the perception of this role from the multidisciplinary team.

## 2. Setting and Sample

The setting of this study is a primary care facility that is part of a large Midwestern tertiary healthcare center. This primary care practice has developed a multidisciplinary team-based approach to care for patients by integrating providers and staff from pediatrics, internal medicine, and family medicine. It utilizes the patient-centered medical home model (PCMH) which allows increased communication between behavioral health specialists and primary care team members in the clinic by having the entire team colocated. This consolidation of role variation allows the opportunity to assess a broader perspective for this study from a multidisciplinary team [3]. The TTS was a family nurse practitioner who worked in this primary care location for the last three years and recently obtained certification in February 2021. The only specific billing completed by the TTS was if the provider saw a patient for a clinic appointment for tobacco cessation; otherwise, no billing was completed for questions or curbside consultations. The sample for this study included all the primary clinic staff that directly work with patients and included licensed practical nurses, registered nurses, physician assistants, certified nurse practitioners, and medical doctors. The Institutional Review Board at the Mayo Clinic approved this study.

## 3. Design

We conducted an electronic cross-sectional survey to evaluate awareness and perception of the integration of a tobacco treatment specialist into a primary care setting (please see the appendix in the Supplementary Materials (available here). The survey was sent to staff emails with description and purpose of the survey including that it should take just a few minutes to complete, reinforcing the confidentiality of the survey, and that data will be deidentified by the Survey Research Center. In respect to the small sample size, data on age and gender were not completed to increase confidentiality. The survey was sent from December 6, 2021, to January 3, 2022. Reminder emails were sent once weekly during this timeframe. No incentives were given to complete survey. Survey domains on getting basic understanding of the role integration was used to develop survey items and provide content validity. Open-ended questions allowed qualitative data to be obtained.

## 4. Results

60 surveys were administered and 46 were completed which resulted in a 76% survey response rate. The nonresponse rates were evenly distributed amongst the groups with the lowest being from the Doctors of Medicine and Physician Assistants. Basic descriptive statistics was used to analyze the data. Demographic information included role at the primary care site and how long they have been in that current role (see Tables 1 and 2).

85% of the staff knew that there was a tobacco treatment specialist at the primary care site (*n* = 39). Of those 39 that knew there was a TTS provider at that location, prior to having this person on site, only 41% had heard of tobacco treatment specialists. Prior to having a tobacco treatment specialist on site, 59% had never heard about tobacco treatment specialists. 55% (*n* = 22) of staff have utilized the tobacco treatment specialist with direct patient care. Reasons for not utilizing the tobacco treatment specialist included feeling confident in managing tobacco use with patients (9%), specialist was not available when needed (9%), tobacco treatment specialist utilization is not needed in my role (14%), lack of time (9%), and other reasons (59%). Other reasons included the role being new that it is easy to forget the resource, the patient declined, no need for utilization, and lack of knowledge on how to use the role.

With regard to the staff that have used the tobacco treatment specialist (55%), reasons for using the specialist was for referral for follow-up tobacco cessation visit (54%), curbside consultations (21%), medication management (21%), and other reasons (5%). Other reasons included having provider reach out to diabetic patients on panels to assist with tobacco cessation and recommendations in unique patient circumstances. The perception of the value from staff for referrals for follow-up tobacco cessation visits included allowing the patient to return to our clinic for the appointment, allowing a specific visit for the patients to discuss medications and counseling options, consistency with follow-up for patients, allowing the provider to focus on other items during visit, knowing the patient would have specific time to discuss tobacco cessation, starting a cessation plan, knowing that patients are more motivated to stop smoking because they do not have to go elsewhere for the care, and having expert knowledge at the clinic. The value of curbside consultation allows for clarification reasoning, discussion of strategies and problem solving in unique challenging situations, learning something new, medication recommendations, and expert knowledge in an efficient timeframe. The value of medication management allows consistency in the patient treatment plan, beneficial in guiding decisions for care, higher success with stopping smoking when on the right management plan, and guidance on medication availability. Other important values of having the TTS integrated into primary care included having someone who is up to date with resources and knowledge available for discussions, and this allows patients to have increased buy-in to have a tobacco-free lifestyle. All staff strongly agreed that the curbside consultation and medication management were valuable. One staff member neither agreed nor disagreed that the referral for follow-up tobacco cessation was valuable, while the other 20 staff members strongly agreed that the referral for follow-up tobacco cessation was valuable.

Diabetic metrics are an important focus at this primary care site, and tobacco use is one of the measurements to drive high-quality metrics. The question focused on individual providers' own personal panel of diabetic patients and the improvement they have perceived for their own metrics. The tobacco treatment specialist improved diabetic quality metrics in 32% of those surveyed, 18% stated no improvement in quality metrics, and 50% of those surveyed did not have diabetic metrics to be measured. Patients mentioned to staff different advantages of discussing nicotine cessation in the primary care clinic as opposed to other nicotine treatment locations which included location/parking (23%), convenience of scheduling (18%), comfort or familiarly with clinic staff such as rooming nurses, front desk (18%), feeling confident with their primary care provider giving personal recommendations (17%), having video visits as an option (1%), and no mention of advantages (23%).

The open-ended questions allowed us to get themes and comments from the questions which were compiled. Comments on the survey about any additional benefits of having the tobacco treatment specialist on site is the ability to ask questions directly with immediate feedback. The income for the clinic and with smaller patient loads can benefit patients for continuity and the one-on-one feeling of care. The ability to have a resource on hand that specializes in tobacco is a great resource for consultations. The workspace design at the primary care site allows for the interaction with specialists which is helpful. Patients appreciate not having to go to different locations for specialty care.

## 5. Limitations

A major limitation for this study was that it was only conducted at one primary care location which provides limited data. In addition, this was only the perception based off one tobacco treatment specialist and may influence perception based off personal characteristics. The survey was formulated based on getting an understanding of the TTS role from the primary author which impacts internal validity of the survey design. Generalizability of having a tobacco treatment specialist may be limited to specific primary care practice settings. Despite these limitations, this study shows the positive impact that having a tobacco treatment specialist may have and helps guide direction for further research.

## 6. Discussion

This research study allows primary care practices to think differently about the impact a tobacco treatment specialist can have if they are integrated into the practice. This survey shows that further education and understanding of the tobacco treatment specialist would benefit and enhance the utilization of the specialist throughout the practice. This role understanding may help with referrals and recommendations for utilizing the specialist on site.

Those that have used the specialist on site have found there to be many benefits with allowing immediate feedback for recommendations. This has the potential to increase patient satisfaction with answers to questions immediately with the confidence that they are getting information from someone who is knowledgeable on the topic. Patients, who have long-standing relationships with their primary care provider, are more likely to be receptive to follow-up, if their provider is giving them a personal recommendation. These patients trust their primary care provider's recommendations and may not want to let their provider down, so they will utilize this resource. Another advantage can be having the primary care provider introduce the TTS to the patient while in the clinic, so there is a direct hand-off care which is not feasible in other specialty areas. Research demonstrates that having easy access to tobacco cessation resources and products increases chances of successful quitting.

Additionally, in primary care, contact with nursing is frequent and often a first line of communication. A triad of care for those who are interested in smoking cessation may be beneficial. The care team nurse can meet with the patient while in the clinic or place an initial phone call. The nurse can then complete follow-up phone calls and field smoking cessation questions with greater confidence by collaborating alongside the TTS, thus creating the potential for greater cessation success.

## 7. Conclusion

This study reinforced the positive impact a tobacco treatment specialist can have being integrated into the primary care practice from the perception of the multidisciplinary team. Further research needs to be conducted on the effectiveness of these specialist visits into primary care, surveying the patients, more integration to other primary care locations, and how to provide additional resources to primary care staff on the role of tobacco treatment specialists.

## Figures and Tables

**Table 1:** Role at primary care site.

Field	Percentage of total respondents	Total responses (46)	Total surveyed	Response rate
Licensed practical nurse (LPN)	35%	16	19	84%
Registered nurse (RN)	28%	13	16	81%
Doctor of medicine (MD)	13%	6	10	60%
Advanced practice registered nurse (APRN)	17%	8	10	80%
Physician assistant (PA)	7%	3	5	60%

**Table 2:** Time at current role.

Time frame	Percentage	Total (46)
Less than 2 years	20%	9
3-5 years	33%	15
6-10 years	33%	15
More than 11 years	15%	7

## Data Availability

The data used to support the findings of this study are included within the article.

## Supplementary Materials

Supplementary Materials Appendix: electronic cross-sectional survey to evaluate awareness and perception of the integration of a tobacco treatment specialist into a primary care setting.
