# Supplementary material for: Integration of a Tobacco Treatment Specialist into Primary Care: Perception from Multidisciplinary Team
Source: J Smok Cessat. 2022 Jun 18;2022:9330393. doi: 10.1155/2022/9330393 (PMC9233593; doi:10.1155/2022/9330393)
Supplement: Supplementary Materials — Appendix: electronic cross-sectional survey to evaluate awareness and perception of the integration of a tobacco treatment specialist into a primary care setting. [file 9330393.f1.docx]

APPENDIX

Q1 **What is your role at your primary care site?**

- Licensed Practical Nurse (LPN) (1)
- Registered Nurse (RN) (2)
- Doctor of Medicine (MD) (3)
- Advanced Practice Registered Nurse (APRN) (4)
- Physician Assistant (PA) (5)

Q2 **How long have you been in your current role?**

- Less than 2 years (1)
- 3 to 5 years (2)
- 6 to 10 years (3)
- More than 11 years (4)

Q3 **Is there a tobacco treatment specialist at your primary care site?**

- Yes (1)
- No (2)
- Don't know (3)

Q4 **Prior to having a tobacco treatment specialist on site, had you heard about tobacco treatment specialists?**

- Yes (1)
- No (2)

Q5 **Have you utilized the tobacco treatment specialist at your site with direct patient care?**

- Yes (1)
- No (2)

Q6 **Which of the following, if any, are reasons why you have not utilized the tobacco treatment specialist at your site?** (Mark all that apply.)

- I feel confident in managing tobacco use with my patients (1)
- The specialist was not available when needed (2)
- Tobacco treatment specialist utilization is not needed in my role (3)
- Lack of time (4)
- Other, please specify: (5) ________________________________________________

Q7 **What was the tobacco treatment specialist used for?**  (Mark all that apply.)

- Referral for follow up tobacco cessation visit (1)
- Curb side consults (2)
- Medication management (3)
- Other, please specify: (4) ________________________________________________

Q8 **What was the value of the referral for follow up tobacco cessation visit interaction, if any?**

Q9 **What was the value of the curb side consults interaction, if any?**

Q10 **What was the value of the medication management interaction, if any?**

Q11 **What was the value of the other interaction, if any?**

Q12 **Please indicate how much you agree or disagree with the following statement(s) about the utilization of the tobacco treatment specialist.**

|  | Strongly agree (1) | Somewhat agree (2) | Neither agree nor disagree (3) | Somewhat disagree (4) | Strongly disagree (5) |
| --- | --- | --- | --- | --- | --- |
| Display This Choice:  If Q7 = 1  The referral(s) for follow up tobacco cessation visit was valuable. (1) |  |  |  |  |  |
| Display This Choice:  If Q7 = 2  The curb side consult(s) was valuable. (2) |  |  |  |  |  |
| Display This Choice:  If Q7 = 3  The medication management was valuable. (3) |  |  |  |  |  |

Q13 **Has the tobacco treatment specialist improved your diabetic quality metrics?**

- Yes (1)
- No (2)
- NA (3)

Display This Question:

If Q3 = 1

Q14 **Have your patients mentioned any of the following advantages to discussing nicotine cessation in the primary care clinic as opposed to other nicotine treatment locations?** (Mark all that apply.)

- Location/parking (1)
- Convenience of scheduling (2)
- Comfort of or familiarity with clinic staff such rooming nurses, front desk (3)
- Feel confident with their primary care provider giving personal recommendation (4)
- Other, please specify: (5) ________________________________________________
- None (6)

Display This Question:

If Q3 = 1

Q15 **Please describe any additional benefits of having the tobacco treatment specialist onsite.**

Q16 THANK YOU FOR COMPLETING THE SURVEY!

 **Please click SUBMIT to record your answers.**
